# Seasonal differences in the effects of oscillatory and uni‐directional flow on the growth and nitrate‐uptake rates of juvenile *Laminaria digitata* (Phaeophyceae)

**DOI:** 10.1111/jpy.12348

**Published:** 2015-10-23

**Authors:** Louise T. Kregting, Christopher D. Hepburn, Graham Savidge

**Affiliations:** ^1^ School of Planning, Architecture and Civil EngineeringQueen's University Marine Laboratory12‐13 The StrandPortaferryBT22 1PFUK; ^2^ Department of Marine ScienceUniversity of OtagoPO Box 56Dunedin9054New Zealand; ^3^ School of Biological SciencesQueen's University Marine Laboratory12‐13 The StrandPortaferryBT22 1PFUK

**Keywords:** kelp, macroalgae, nitrogen, productivity, water motion

## Abstract

The influence of oscillatory versus unidirectional flow on the growth and nitrate‐uptake rates of juvenile kelp, *Laminaria digitata*, was determined seasonally in experimental treatments that simulated as closely as possible natural environmental conditions. In winter, regardless of flow condition (oscillatory and unidirectional) or water velocity, no influence of water motion was observed on the growth rate of *L. digitata*. In summer, when ambient nitrate concentrations were low, increased water motion enhanced macroalgal growth, which is assumed to be related to an increase in the rate of supply of nutrients to the blade surface. Nitrate‐uptake rates were significantly influenced by water motion and season. Lowest nitrate‐uptake rates were observed for velocities <5 cm · s^−1^ and nitrate‐uptake rates increased by 20%–50% under oscillatory motion compared to unidirectional flow at the same average speed. These data further suggested that the diffusion boundary layer played a significant role in influencing nitrate‐uptake rates. However, while increased nitrate‐uptake in oscillatory flow was clear, this was not reflected in growth rates and further work is required to understand the disconnection of nitrate‐uptake and growth by *L. digitata* in oscillatory flow. The data obtained support those from related field‐based studies, which suggest that in summer, when insufficient nitrogen is available in the water to saturate metabolic demand, the growth rate of kelps will be influenced by water motion restricting mass transfer of nitrogen.

**Abbreviations**
PFD: Photon flux density
RGR: Relative growth rates

The availability of nutrients, particularly nitrogen, is a key factor influencing the growth and productivity of macroalgae (e.g., Hanisak 1983, Lobban and Harrison 1997, Fong 2008). The supply of nutrients to the macroalgal thallus affects physiological and biochemical processes (Wheeler 1980, Hurd 2000) that can reduce primary productivity particularly during periods of low seawater nitrogen concentration (Hepburn et al. 2007, Stephens and Hepburn 2014). The rate of nutrient‐uptake by a macroalgal thallus depends not only on the external concentration of the nutrient, but also on the nutrient quota of the macroalgae (e.g., McGlathery et al. 1996) and the rate of supply of nutrients from the water immediately adjacent to the blade surface as mediated by water motion (Hurd 2000). Water motion influences nutrient availability and gas exchange owing to its effect on boundary layer formation, a region of reduced velocity at the thallus surface. The dynamics of boundary layer formation control the rate at which nutrients are delivered to the cell and hence dictate the rate of uptake of the nutrients into the cell, a phenomenon termed “mass‐transfer limitation” (Hurd 2000).

It is reasonable to say that our present understanding of the effects of water velocity on nutrient‐uptake rates and photosynthesis of macroalgae is primarily based on experiments where macroalgae are subjected to a unidirectional flow‐field. These experiments demonstrate that rates of nutrient‐uptake (Hurd et al. 1996, Kregting et al. 2008a), photosynthesis (Carpenter et al. 1991, Gonen et al. 1993) and growth rate (Parker 1981, 1982, Fujita and Goldman 1985) increase with increasing water velocity until a saturating velocity is reached (2.5–6 cm · s^−1^; Wheeler 1980, Gerard 1982). Data from these studies can be applied to the upper subtidal and lower littoral zones where macroalgae are subjected to unidirectional flow predominantly associated with tidally driven currents, especially in inlets, harbors and estuaries (Wright et al. 1999). In such current‐dominated locations it is not uncommon for macroalgae to experience flow rates <6 cm · s^−1^ for a defined period of time during a tidal cycle (Kregting et al. 2008b, 2011). Low flow rates or stagnant conditions may also occur in rock pools during slack tides or within dense canopy‐forming macroalgae (Kregting et al. 2011) resulting in macroalgae frequently experiencing mass‐transfer limitation on timescales of minutes to hours.

It is not clear if evidence from experiments using uni‐directional flow fields provides a reasonable basis for the prediction of responses of macroalgae to marked oscillatory ambient water motion as will result from the influence of wind and swell‐driven waves on open coasts (Denny and Gaylord 2002). Very little is known about the effects of oscillatory flow on the physiological processes of macroalgae. For seagrass and algal turfs, both nutrient‐uptake rates and productivity have been demonstrated to be higher in oscillatory as opposed to unidirectional flow (Carpenter et al. 1991, Thomas and Cornelisen 2003). These observations were attributed to a reduction in the diffusion boundary layer (DBL) thickness associated with increased turbulence at the blade surface under oscillating flow conditions (Stevens and Hurd 1997). Continual reversals in the flow direction increase the turbulence at the blade surface with the result that at similar relative flow velocities, boundary layers will be thinner in oscillatory motion than unidirectional flow (Denny 1988). Observations of higher growth rates and nitrogen contents for the giant kelp *Macrocystis pyrifera* in wave‐exposed compared to current‐dominated sites (Hepburn et al. 2007) suggests that the advantages for uptake of nitrogen provided by oscillatory flow observed in algal turfs and seagrass may also hold true for larger kelp species.

Any advantage provided by flow type and/or flow speed on macroalgal physiology and growth or nutrient‐uptake rates is likely to be more pronounced when macroalgae have a high metabolic demand (Hepburn et al. 2007, Stephens and Hepburn 2014). Such conditions could be associated with periods of increased growth or at times of low internal nitrogen status. This has been observed for the kelp *M. pyrifera*, where exposure to a marked wave‐field modified the seasonal pattern of growth by ameliorating low seawater nitrogen concentrations during summer and autumn, when nutrients were available in the water column at moderate concentrations and metabolic demand was high (Hepburn et al. 2007).

Thie present study examined the effect of oscillatory versus unidirectional flow on the growth and nitrate‐uptake rates of juvenile kelp, *Laminaria digitata* (Hudson) J.V.Lamouroux between the summer period when metabolic demand for nitrogen is high and the winter period when *L. digitata* is nitrogen replete. The experimental treatments used were designed to simulate as closely as possible natural environmental conditions. The investigation had two complementary objectives: (i) to assess the influence of steady and oscillating flow regimes on the nutrient‐uptake and growth rates of juvenile *L. digitata* and (ii) to establish whether seasonal differences in metabolic demand for nitrogen influenced this relationship.

## Materials and Methods

### Oscillatory and unidirectional tank design and experimental setup

Nutrient‐uptake and growth rates of the juvenile kelp *L. digitata* (~25 cm length and ~2.5 g wet weight) were determined in experimental culture‐tank systems designed to simulate both oscillatory and unidirectional water motion. The design of the culture tanks was developed to address inherent engineering difficulties in developing standard flume technology to mimic oscillatory flow on the size scale required for this study, where it was necessary to replicate the oscillatory and unidirectional water motion experiments simultaneously. In the culture tanks, kelp individuals were moved through a stationary body of water to simulate oscillatory and unidirectional water motion representative of the wave and current‐dominated regimes rather than the typical approach of moving water past a fixed organism (Hurd et al. 1994, Kregting et al. 2008a, 2013).

**Figure 1:**
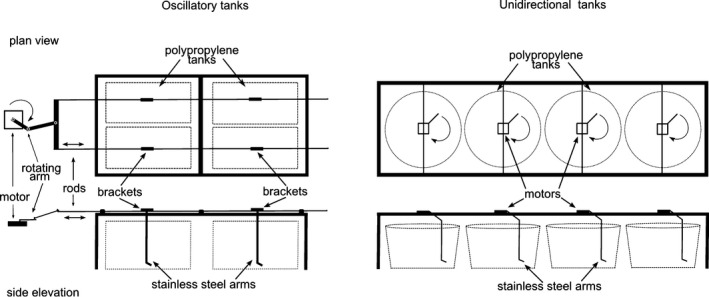
Diagram of the experimental oscillatory and unidirectional tanks. The tanks were used to assess the effects of oscillatory or unidirectional flow on the growth and nitrate‐uptake rates of juvenile kelp *Laminaria digitata*.

The horizontal oscillatory motion that organisms are subjected to, close to the sea bed, was simulated in laboratory experimental tanks by fixing two rods on brackets that were mounted above the culture tanks on a steel frame allowing free oscillatory movement of the rods. The rods were attached to a rotating arm driven by a 12 V car windscreen wiper motor (Fig. 1). Two stainless steel arms were attached to each rod (arms = 4). Each arm, which could be easily disconnected from the rods, was used to attach individual juvenile kelps by their holdfast and was suspended in one of four replicate 65 L capacity square white polypropylene containers (60 × 40 × 32 cm). To simulate unidirectional flow, a separate frame was built with each stainless steel arm attached to an individual 12 V windscreen wiper motor which had been modified to provide a steady rotatory movement in each of four replicate 60 L capacity circular white polypropylene experimental tanks (58 D × 33.5 H cm; Fig. 1). As it was necessary to reduce the volume of water in the tanks used for nutrient‐uptake rate determinations in the unidirectional flow experiments, smaller tanks with a 24 L capacity (35 D × 25 H cm) were placed inside the 60 L circular tanks. This allowed enough space for each arm to move freely, while ensuring that each individual kelp plant was adequately covered with water.

All drive motors used to generate the appropriate current field were powered by regulated power supplies (Skytronic 0–30 V; Onecall, Leeds, UK). Water velocities within the culture tanks could not be measured by standard methods such as Acoustic Doppler Velocimeters (Kregting et al. 2011). Thus, flow speeds were calculated by adjusting the voltage of the power supplies and measuring the distance the arms moved over a period of time. Water velocities were replicated in each of the four oscillatory experimental tanks as a single motor was used to drive all four arms to which the kelps were attached. However, a separate drive motor was used for each of the four unidirectional flow tanks; despite the same drive voltage being used for each motor, each showed some speed variation. To accommodate this variability, the oscillatory and unidirectional flow treatments were placed into flow velocity groups as follows: 0–4.99, 5–9.99, 10–14.99, 15–22 cm · s^−1^.

During the growth measurement experiments, each tank was supplied with flow‐through running seawater from the laboratory seawater system supplied from the Strangford Narrows to maintain as close as possible ambient temperature and nutrient concentrations. The seawater was supplied to the tanks at a low flow rate (~1 L · min^−1^) to ensure minimal interference with flow hydrodynamics around the individual kelps.

All experiments were carried out in a room where temperature was maintained at ~10°C–12°C over a year. Photon flux density (PFD) was supplied by cool white 70 W fluorescent lights (Fitzgerald Lighting Cornwall, Cornwall, UK) on a 12 h:12 h LD cycle. Using a cosine corrected quantum sensor (Skye SKP 215; Llandrindod Wells, Powys, UK), PFD was adjusted so that individuals received 20–30 μmol photons · m^−2^ · s^−1^ (0.9–1.3 mol photons · m^−2^ · d^−1^) at the blade surface. These irradiance levels have been shown to saturate growth of juvenile *L. digitata* inhabiting low light regions under the adult canopy (Kain 1965, Han and Kain 1996) and are within the range known to saturate growth in adult sporophytes (Bartsch et al. 2008).

### Determination of blade growth rates

Growth rates of the blades of juvenile *L. digitata* thalli under oscillatory and unidirectional flow conditions were determined over two separate 6 week periods in winter (November 20, 2009 to January 2, 2010) and summer (June 24 to August 5, 2010). As only four replicates for each experimental flow velocity group could be carried out in the unidirectional tanks (*n* = 4) and oscillatory tanks (*n* = 4), each week represented a different flow velocity group which was randomized. Tanks were emptied between each experiment and cleaned with freshwater. Preliminary experiments showed no difference in kelp growth rates when juveniles were placed in the experimental tanks for a total of either 7 or 14 d and hence each growth experiment was run for 7 d in order to minimize ambient changes in temperature and seawater nutrients during the winter and summer 6 week experimental periods.

Juvenile *L. digitata* individuals used in the experiments were collected from the low intertidal region in the Strangford Narrows at the entrance to Strangford Lough, Northern Ireland (N 54° 22.903, W 005° 33.241). Thalli <30 cm in length, with an approximate wet weight between 2 and 2.5 g · wwt^−1^, were gently removed from the substrate with their holdfast intact during low water on the day before experimentation and transported the short distance to the laboratory. Silt and any epiphytes were removed by gently wiping the blades with tissue while material was removed from the holdfast using forceps.

The blades of the juvenile *L. digitata* were too small to use the standard hole‐punch method of Parke (1948) to estimate growth. Thus, a method adapted from Kregting et al. (2008b) was used in which a needle threaded with nylon was inserted ~3 cm from the stipe/lamina transition zone, the thread tied off and growth measured from the increase in distance of the marker from the transition zone. Following marking, the thalli were placed in similar light and temperature conditions as used for the experiments to allow recovery from any stress induced from handling and marking. For each experimental week (7 d), initial and final measurements of the meristem region (stipe/lamina transition zone to the threaded hole) were made on each juvenile kelp and surface area estimated from digital images using Image‐Pro^®^ Plus (Media Cybernetics, version 1.47 for Windows^™^, Rockville, MD, USA). However, it was not possible to use the surface area data for every blade owing to evidence of distal blade erosion. Relative growth rates (RGR) based on increases in the length of blade between the threaded hole and the transition zone and thallus surface area were determined after Evans (1972) using the following equation:

(1) $$\text{RGR}=\;\frac{\ln\left(L_{f}\right)-\ln\left(L_{i}\right)}{T}$$

where *L*_i_ is the initial length between the threaded hole and transition zone (mm)or surface area (cm^2^), *L*_f_ is the final length in distance between the threaded hole and transition zone (mm) or surface area (cm^2^) and *T* is the number of days between measurements (d). The two parameters provide estimates of the maximum growth rate by the meristematic region as well as a growth rate based on the increase of the material in the whole blade. Once the initial measurements were carried out, one individual was placed into a randomly selected tank (*n* = 8) by tying the holdfast to the arm using cotton so that the juvenile was stabilized in an upright position for the duration of the experiment (7 d).

Samples of seawater (3 × 60 mL) were taken at the start of each experimental week, filtered (Whatman GF/C glass‐fiber filters, GE Healthcare UK Limited, England, UK) and frozen at −20°C for later analysis of nutrient concentrations to determine changes in concentrations over the course of the experiment. The samples were analyzed for nitrate (NO_3_^−^), phosphate (PO_4_^3−^) and ammonium (NH_4_^+^) concentrations using standard colorimetric procedures with a Bran+Luebbe AutoAnalyzer 3 (Seal Analytical Ltd, Fareham, UK). Seawater temperature was also noted with a thermometer at the beginning of each experimental week. To eliminate any potential bias with ambient environmental variables, flow velocity treatments were randomized over the experimental period.

### Estimation of nutrient‐uptake rates

Nutrient‐uptake rates of the *L. digitata* juvenile thalli were determined for each of the four flow velocity groups over five consecutive days in summer (August 2010) and winter (February 2011). As with the growth experiments, entire thalli of the ~15 randomly selected juvenile *L. digitata* individuals used for nutrient‐uptake experiments and tissue nutrient analysis, were collected prior to each experiment from the Strangford Narrows site, cleaned and placed in conditions similar to those to be used for the uptake rate measurements. At the start of each experiment, the tanks were filled with 20 · L seawater from the laboratory supply with no added water supply for the duration of the experiment. The seawater had been analyzed the previous day for nitrate concentration. An initial addition of nitrate (KNO_3_) to provide an experimental nitrate concentration of 5 μM was required for the summer experiment. Ammonium uptake rates were not estimated as ambient ammonium concentrations in the laboratory seawater supply had been shown to be undetectable.

For each tank, the seawater was mixed thoroughly and three initial (*i*) 10 mL seawater samples were removed for nutrient analysis. For each tank, one juvenile *L. digitata* was selected from the pre‐experimental holding tank and attached to the stainless steel arm by tying the holdfast with cotton and placing it randomly in a tank (*n* = 8). The motors were then set at randomized pre‐determined experimental flow speeds for 6 h, after which the juveniles were removed and dried at 80°C for 48 h. The seawater in the tanks was again mixed thoroughly and three final (*f*) seawater samples were taken from each tank for subsequent analysis of nitrate concentration as above.

Preliminary experiments were carried out to determine the incubation time required for each nutrient‐uptake rate experiment to ensure a detectable (>0.2 μM) change in seawater nutrient concentration (Hurd et al. 1994). Seawater temperature was also noted at the beginning and end of these preliminary trials. An increase <2°C for each tank was recorded and although this may have some influence on nutrient‐uptake rates, the increase was consistent between experimental treatments and days.

Control experiments without algae were conducted at the highest flow velocity group to determine changes in nutrient concentration in the experimental tanks in the absence of the kelps over 6 h. The use of these controls was important as it was not possible to pre‐filter the seawater supplied to the culture tanks owing to the large volume of seawater required.

Nutrient‐uptake rates for nitrate (*V*, μmol [g dwt]^−1^ · h^−1^) for each experimental flow condition were calculated using the following equation:

(2) $$V=\frac{S_{i}-S_{f}}{t\times \text{dwt}}\times \text{vol.}$$

where *S*_i_ is the initial substrate concentration (μM), *S*_f_ is the final substrate concentration (μM), vol. is the volume of the tank (*L*), t is time (h), and dwt is dry weight (g).

### Determination of tissue nutrient concentrations

Soluble tissue nitrate and ammonium concentrations were determined during the growth and nutrient‐uptake rate experiments. At the start of each growth experimental week and on day 1, 3 and 5 of the nutrient‐uptake experiments, five juvenile kelps with similar sizes as the experimental kelps were haphazardly selected from the collection site. Two small disks (2 cm diameter) were punched from the blade side by side ~1 cm from the stipe/lamina junction, gently cleaned of any sediment or epiphytic material and wiped and blotted dry for wet weight determination. The disks were trimmed to equal weight with one disk dried at 80°C for 48 h. The other disk was analyzed for soluble nutrient pools using the boiling‐water extraction method of Hurd et al. (1996). Preliminary analyses showed that boiling the sample twice was sufficient to remove all soluble nutrients. Extracted samples were stored in 60 mL polyethylene bottles and frozen (−20°C) for later analysis.

### Data analyses

Comparison of the meristematic blade RGR, increase in surface area RGR (*n* = 3–9) and nutrient‐uptake rates (*n* = 3–8) of juvenile *L. digitata* as a function of flow velocity groups (0–4.99, 5–9.99, 10–14.99, 15–22 cm · s^−1^), tank (oscillatory and unidirectional) and season (winter and summer) were carried out using a three‐way ANOVA (general linear model). A two‐way ANOVA was used to test for differences in seawater nitrate concentration (*n* = 3) and soluble tissue ammonium and nitrate pools (*n* = 5) among experimental days/weeks within and between seasons. Significant differences between treatments were compared using Tukey's honestly significantly different (HSD) and Fisher's least significant difference (LSD) post‐hoc tests. Levene's test for homogeneity of variances and the Kolmogorov–Smirnov test of normality were carried out and, where necessary, data were log transformed. All analyses were performed using IBM SPSS 19.0 for Windows^©^ 2010 (Portsmouth, Hampshire, UK). A significance level of *P* ≤ 0.05 was used for all statistical tests.

## Results

### Growth experiments

**Figure 2:**
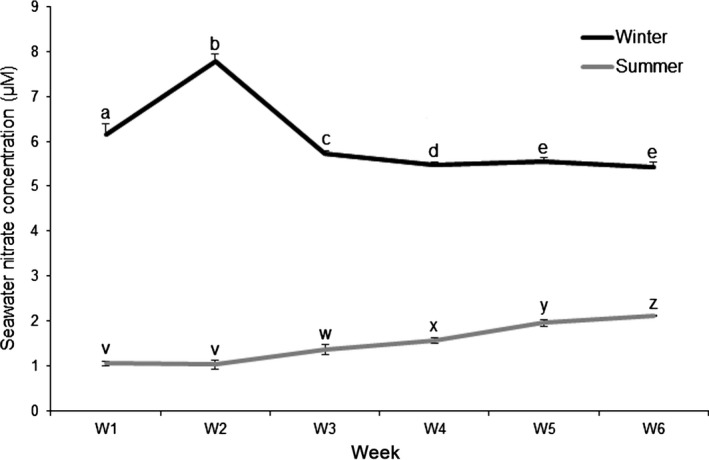
Ambient seawater nitrate concentrations over each 6‐week experimental growth period in winter (November 20, 2009 to January 2, 2010) and summer (June 24 to August 5, 2010). Means ± SE (*n* = 3). Dif‐ferent letters denote significant differences in seawater concentra‐tions between weeks within each season: winter (a, b, c, d, e) and summer (v, w, x, y, z) (LSD,* P* < 0.05).

The temperature of the seawater supplied to the experimental tanks varied over each 6‐week experimental period by ~3°C (9.0°C–11.9°C) in winter and ~2.5°C (12.8°C–14.3°C) in summer. Variation in seawater nitrate concentrations during the 6‐week experimental period were also observed (Table 1, Fig. 2) with concentrations varying ~3 μM in winter and ~1.3 μM in summer. Nitrate concentrations within the experimental tanks were higher in winter (6.2 μM) than summer (1.5 μM; Table 1; Fig. 2). Phosphate concentrations were stable during the 6‐week experimental period in winter (0.9 ± SE 0.03 μM) and summer (0.4 ± SE 0.01 μM; data not shown). Seawater ammonium concentrations were undetectable throughout the duration of the study.

**Table 1:** Results of two‐way ANOVA for seawater nitrate concentration and soluble tissue nitrate and ammonium (ln nitrate and ln ammonium) determined during the growth experiments of juvenile *Laminaria digitata* as a function of week (1–6) and season (winter and summer)

	*F*	df	*P*
Seawater nitrate			
Week	125.63	5, 24	<0.001
Season	44544.82	1, 24	<0.001
Week × season	555.79	5, 24	<0.001
Nutrient pools (ln nitrate)			
Week	9.09	5, 46	<0.001
Season	59.45	1, 46	<0.001
Week × season	3.69	5, 46	<0.001
Nutrient pools (ln ammonium)			
Week	8.78	5, 47	<0.001
Season	0.49	1, 47	0.483
Week × season	15.63	5, 47	<0.001

**Figure 3:**
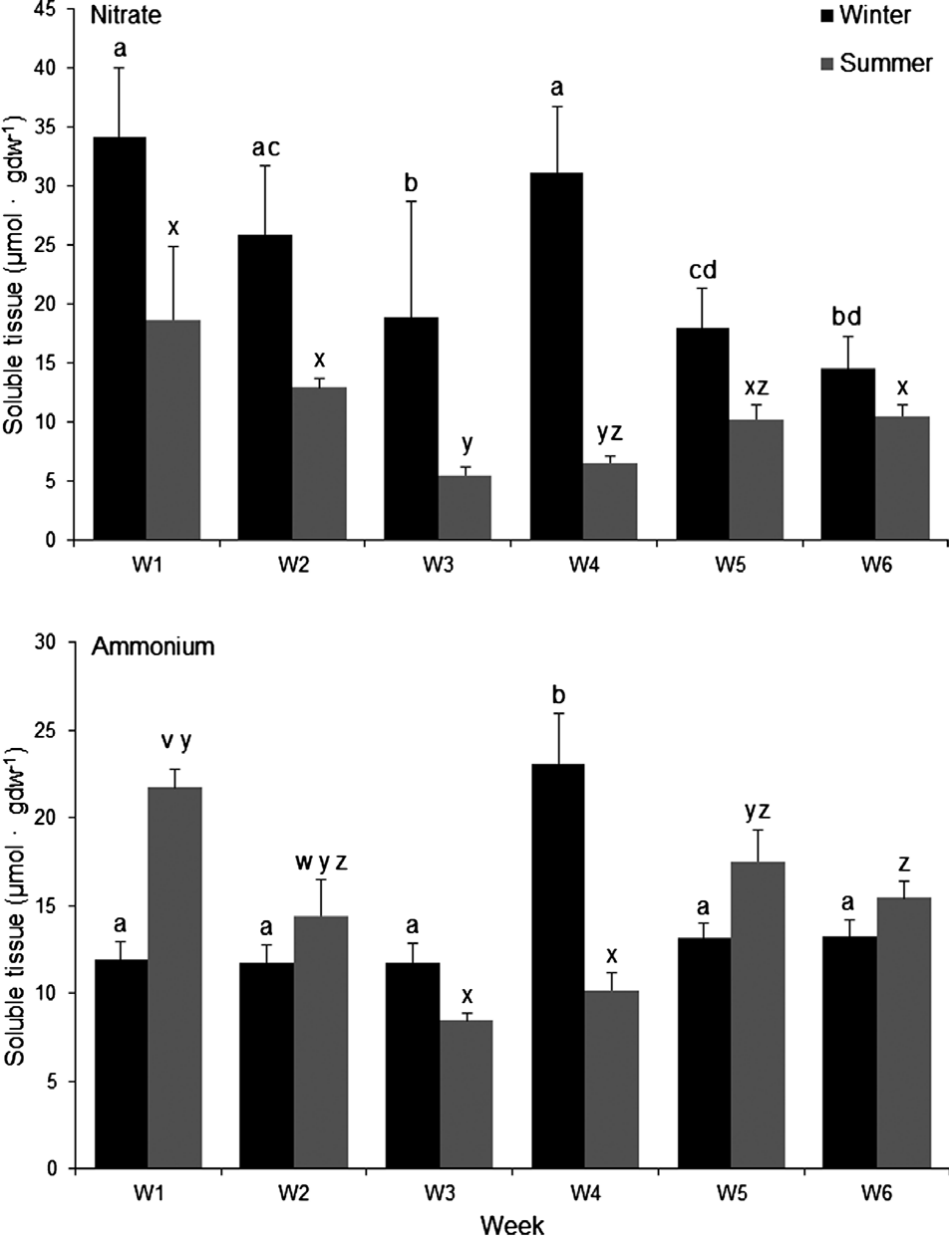
Soluble tissue concen‐trations of nitrate and ammo‐nium of juvenile *Laminaria digitata* blade tissue during each 6‐week experimental growth period in winter (November 20, 2009 to January 2, 2010) and summer (June 24 to August 5, 2010). Means ± SE (*n* = 5). Different letters denote signifi‐cant differences in soluble tissue concentrations of nitrate and ammonium between weeks within each season: winter (a, b, c, d) and summer (v, w, x, y, z) (LSD,* P* < 0.05).

Soluble nitrate pools of *L. digitata* tissue taken prior to each experimental week were higher in winter than in summer, and reflected seasonal changes in seawater nitrate concentration, while tissue ammonium concentrations did not change with season (Table 1; Fig. 3). There was some variability in both nitrate and ammonium pools among weeks during winter and summer but trends were inconsistent (Table 1; Fig. 3).

### Seasonal blade growth rate and flow conditions

**Figure 4:**
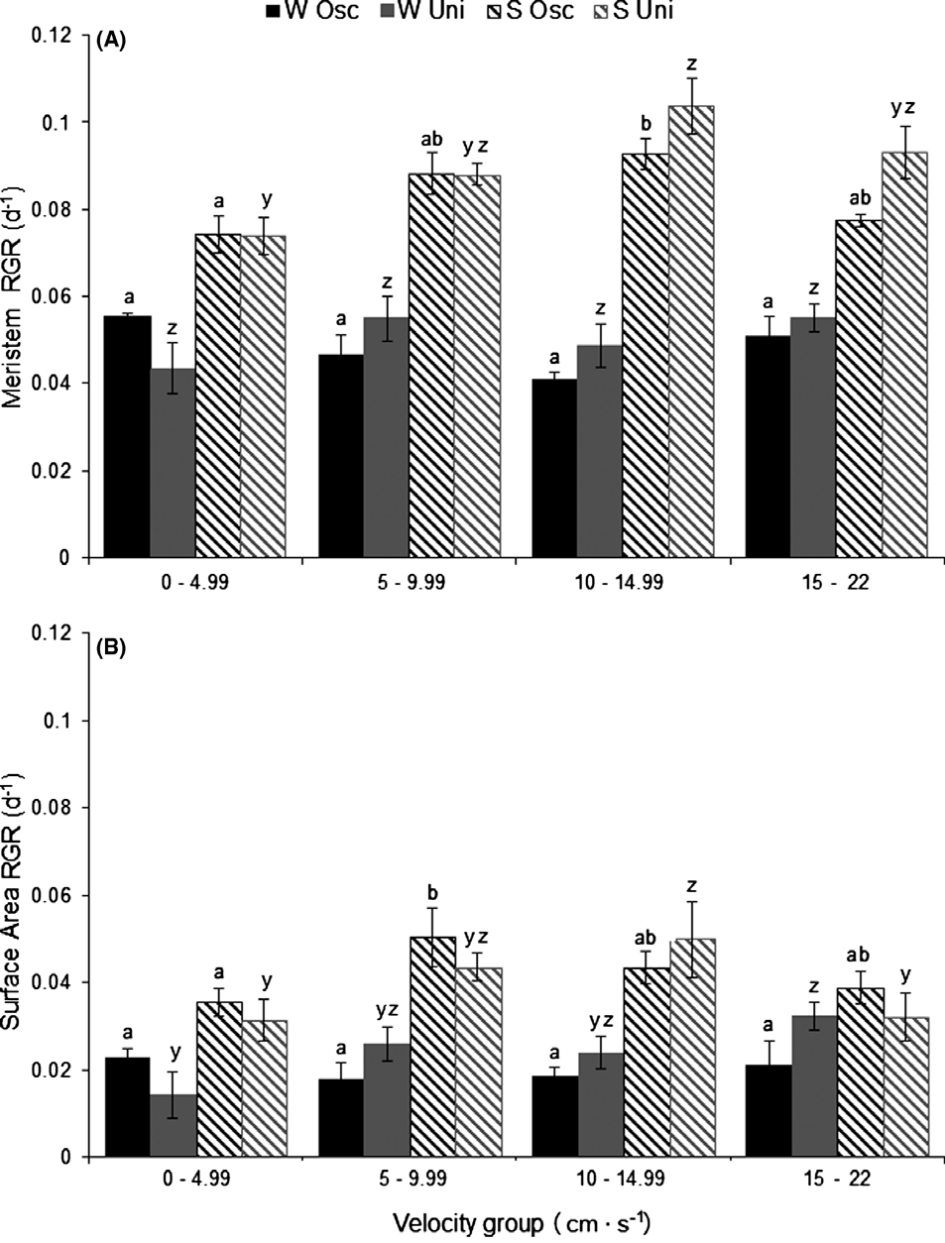
Relative growth rate of the meristematic region (A) and surface area (cm^2^) (B) at four flow velocity groups in oscillatory (Osc) or unidirectional (Uni) flow in winter (W; November 20, 2009 to January 2, 2010) and summer (S; June 24 to August 5, 2010). Means ± SE (*n* = 3–9). Different letters denote signifi‐cant differences in growth rate between velocity categories for oscillatory (a, b) and unidirec‐tional (y, z) flow regimes (LSD,* P* < 0.05). No significant differ‐ences were observed between flow regime (oscillatory and unidirectional).

Meristematic blade RGR and surface area RGR of *L. digitata* juveniles were significantly lower in winter than summer, with the blade meristematic RGR averaging 0.05 d^−1^ in winter compared to 0.08 d^−1^ in summer and surface area RGR increase averaging 0.02 d^−1^ in winter and 0.05 d^−1^ in summer (Table 2; Fig. 4). The interaction of velocity and season was significant for both meristematic blade and surface area growth of *L. digitata* (Table 2). Pair‐wise comparison tests indicated that velocity had an effect on meristematic RGR in summer, but not in winter, with the greatest differences in summer being observed between velocity groups 0–4.99 and 10–14.99 cm · s^−1^ (Fig. 4A). A similar scenario was observed for surface area RGR with pair‐wise comparison tests indicating that velocity had an effect in summer on growth and only in winter for the unidirectional tanks. Depending on flow velocity group, velocity group 5–9.99 and 10–14.99 cm · s^−1^ showed differences with 0–4.99 and 15–22 cm · s^−1^ (Fig. 4B). There was no significant difference observed between oscillatory or unidirectional flow conditions and at any of the velocity categories in both winter and summer (Table 2; Fig. 4).

**Table 2:** Results of a three‐way ANOVA for meristematic and surface area growth rates of juvenile *Laminaria digitata* as a function of flow velocity groups (0–4.99, 5–9.99, 10–14.99, 15–22 cm · s^−1^), flow regime (oscillatory and unidirectional), and season (winter and summer)

	Meristem			Surface area		
	*F*	df	*P*	*F*	df	*P*
Velocity	1.601	3, 74	0.196	2.682	3, 72	0.053
Season	130.564	1, 74	<0.001	60.021	1, 72	<0.001
Flow regime	1.740	1, 74	0.191	0.048	1, 72	0.828
Velocity × season	3.008	3, 74	0.036	3.076	3, 72	0.033
Velocity × flow regime	1.195	3, 74	0.318	1.013	3, 72	0.392
Season × flow regime	0.479	1, 74	0.491	1.966	1, 72	0.165
Velocity × season × flow regime	0.683	3, 74	0.566	1.399	3, 72	0.250

### Nutrient‐uptake experiments

Seasonal differences in ambient seawater temperatures were observed between experiments conducted in winter and summer with average values of 8.5°C recorded in winter and 14°C in summer. Soluble tissue nitrate and ammonium concentrations of *L. digitata* prior to experiments were significantly higher in winter (nitrate 51.6 ± 3.5 and ammonium 17.3 ± 0.8 SE μmol · g · dw^−1^) than in summer (nitrate 9.8 ± 0.5 and ammonium 15.5 ± 0.7 SE μmol · g · dw^−1^; Table 3). Some variability in soluble tissue ammonium was observed between days, but only in summer (pair‐wise comparison *F*_2,24_ = 11.29, *P* < 0.001) varying by ~3.5 (14.5–18.0 μmol · g^−1^ · dw^−1^). There was no variability between experimental days for soluble tissue nitrate in winter or summer (Table 3).

**Table 3:** Results of two‐way ANOVA for soluble tissue nitrate and ammonium determined during the nutrient‐uptake experiments of juvenile *Laminaria digitata* as a function of days (*n* = 3) and season (winter and summer)

	*F*	df	*P*
Nutrient pools (nitrate)			
Day	0.28	2, 22	0.756
Season	600.76	1, 22	<0.001
Day × Season	2.89	2, 22	0.07
Nutrient pools (ammonium)			
Day	5.64	2, 24	0.01
Season	4.95	1, 24	0.036
Day × season	8.71	2, 24	0.001

**Figure 5:**
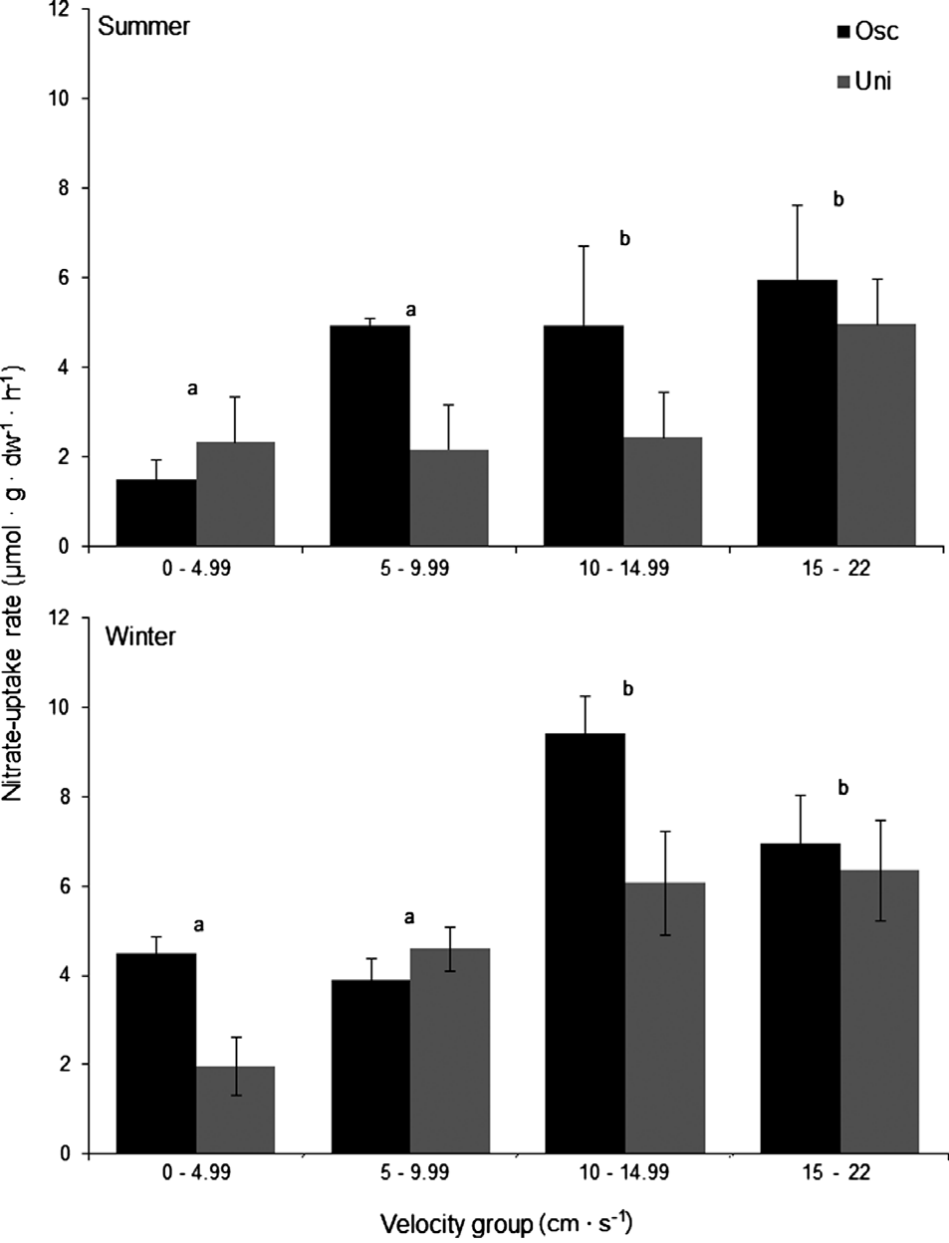
Nitrate‐uptake rates of juvenile *Laminaria digitata* at four flow velocity groups in oscillatory (Osc) or unidirectional (Uni) flow in summer (August 2010) and winter (February 2011). Means ± SE (*n* = 3–8). Different letters denote significant differ‐ences in nitrate‐uptake rates between flow velocity groups (Tukey's HSD,* P* < 0.05).

Changes in nitrate concentration in the controls without algae were minimal (<4%) so that changes in nitrate concentration in the tanks containing algae were attributed to uptake by juvenile *L. digitata*. Nitrate‐uptake rates of the juvenile *L. digitata* kelps were lower in summer than in winter (Table 4; Fig. 5). There were also significant differences in nitrate‐uptake rates between velocity categories (Table 4; Fig. 5) with, in general, lowest nitrate‐uptake rates observed for velocities <10 cm · s^−1^ and higher nitrate‐uptake rates for velocities >10 cm · s^−1^. Unlike growth experiments, significant differences in uptake rates were observed between experimental flow regimes with higher nitrate‐uptake observed under oscillatory flow compared to unidirectional flow of a similar magnitude, especially in the summer experimental period (Table 4; Fig. 5).

**Table 4:** Results of a three‐way ANOVA for the nitrate‐uptake rates of juvenile *Laminaria digitata* blade tissue as a function of flow velocity groups (0–4.99, 5–9.99, 10–14.99, 15–22 cm · s^−1^), flow regime (oscillatory and unidirectional) and season (winter and summer)

	*F*	df	*P*
Velocity	9.467	3, 54	<0.001
Season	11.720	1, 54	0.001
Flow regime	7.039	1, 54	0.010
Velocity × season	2.007	3, 54	0.124
Velocity × flow regime	0.906	3, 54	0.444
Season × flow regime	0.007	1, 54	0.934
Velocity × season × flow regime	1.787	3, 54	0.161

## Discussion

Increasing water motion enhanced the growth of juvenile *L. digitata* during the summer but not in winter, a result consistent with that observed in field‐based studies on *M. pyrifera* (Hepburn et al. 2007, Stephens and Hepburn 2014). Oscillatory water motion designed to mimic waves did not increase growth compared to unidirectional flow of a similar magnitude at any time during this study. While growth was not affected, a pattern of higher nutrient‐uptake rate by *L. digitata* was observed in oscillatory motion compared to unidirectional motion of a similar magnitude. Enhanced nitrogen uptake rates due to increased mass transfer resulting from oscillatory flow is proposed as the mechanism that drives increased growth by kelp in wave‐exposed sites (Hepburn et al. 2007).

In the experiments presented here, the mechanism of increased nitrogen uptake in oscillatory flow is clear but it appears growth is disconnected from any enhanced nitrogen uptake associated with this effect. We speculate that the week‐long experimental period was insufficiently long to allow a significant response in growth relating to flow type to be demonstrated. Increased flow velocity would appear to be the primary driver of growth (Parker 1981, 1982), and that more time may be required to see any additional enhancement of growth by the secondary influence of flow type. It is also possible that in oscillatory flow the kelp are investing more energy into structural compounds to ensure they are not displaced from the rocks by wave action thus influencing growth based on length and area. A secondary response of kelp blades to physical stress is the balance in metabolic cost associated with increasing the thickness of the blade to strengthen against potential damage as opposed to directing resources into lengthening of the blades (Demes et al. 2011). As more nutrients become available under oscillatory motion, the kelp may be consolidating and thickening tissue rather than growing in length in response to increased physical stress. The growth strategy of kelps in relation to the strengthening of cell tissue to ameliorate physical damage versus blade growth has been discussed as a possible explanation for lower blade growth rates of interior *M. pyrifera* individuals, located closest to rocky shores (Stephens and Hepburn 2014). It has been observed that when *M. pyrifera* was provided extra nutrients in the field, blade erosion decreased (Stephens and Hepburn unpub.).

Distal blade erosion was observed in this study at the highest flow rates in some of the blades in both seasons suggesting that physical stress was occurring. As indicated previously, data from these individuals were not included in the growth rate data based on surface area analysis. Potentially we could have addressed the question of blade strength by making wet weight estimates of the experimental juvenile blades at the start and end of the experimental week. However, initial application of this approach showed an unacceptable level of variability in the data. This was associated with the rapidity with which the moisture content components of the wet weight of the thin, delicate blades changed owing to local variability in ambient laboratory conditions. As a consequence, it was not possible to develop SA:WW data to assess whether blades subjected to higher flow rates were characterized by increased tissue thickness.

One of the key limitations of a replicated laboratory‐based study is the inability to build a kelp assemblage. Kelp are found in beds with each individual modifying the hydrodynamic environment of the other (Gaylord et al. 2007, Kregting et al. 2011). In strong unidirectional flow, skimming flows can develop, reducing mixing between the bulk water and water inside the kelp canopy (Ackerman and Okubo 1993, Koch and Gust 1999). Oscillatory flow results in a dynamic kelp bed reducing these bed scale effects on mass transfer (Koch and Gust 1999). It is possible these bed scale effects are the major driver of enhanced growth in situ at wave‐exposed sites and the reduced influence of flow type in the experiments presented is reflective of this. These results do, however, further reinforce the importance of the DBL in influencing nutrient‐uptake and growth rates, especially in summer (Wheeler 1980, Parker 1981, 1982, Hepburn et al. 2007). It is therefore expected that in kelp populations inhabiting coasts where wave action is generally the most dominant form of motion, the oscillatory flow regime will enhance the mass transport of nutrients to the macroalgal surface. Further work is required to understand how increased mass transfer of nitrogen transfers into growth responses and how trade‐offs between increased structural requirements as well as bed scale processes influences the potential for increased productivity in wave‐exposed environments.

The positive relationship observed between nutrient‐uptake rates by the juvenile *L. digitata* and flow speed is consistent with results previously reported by Gerard (1982), Hurd et al. (1996) and Kregting et al. (2008a). The present data also indicate that, in winter, ambient flow velocities ~10 cm · s^−1^ are high enough to support nitrate demand by the juvenile algae when the growth rate was lower. Comparable data for the summer nitrate‐uptake rates indicate that flow velocities >5 cm · s^−1^ are sufficient to support the nitrate‐uptake rates demand during this time when growth rates were higher, presumably controlled by the endogenous circannual rhythm that *L. digitata* exhibit (Schaffelke and Lüning 1994) as well as increased water temperature (Davison 1991). The summer limiting flow velocities are close to the equivalent velocities of 2.5 cm · s^−1^ for *M. pyrifera*, 6 cm · s^−1^ for *M. integrifolia* and 6 cm · s^−1^ for *Adamsiella chauvinii* reported by Gerard (1982), Hurd et al. (1996), and Kregting et al. (2008a), respectively. These previous data have been interpreted to suggest that the DBL plays a significant role in influencing nutrient‐uptake rates with increasing flow velocities reducing the thickness of the DBL and it is assumed that the present data are consistent with this hypothesis.

Although unidirectional flow may be important in controlling nutrient‐uptake by macroalgae in sheltered coastal environments, kelp beds in wave‐exposed habitats will be subject to oscillatory flow, associated particularly with wind and swell‐driven wave activity. To the best of our knowledge, the present investigation is the first to assess experimentally how oscillatory motion may influence nutrient‐uptake by a large bladed macroalgae. Nutrient‐uptake rates of the juvenile kelps placed in oscillatory flow conditions were between 20% and 50% higher than the juveniles placed in comparable unidirectional flow conditions and were comparable to similar data obtained for seagrass and algal turfs (Carpenter et al. 1991, Thomas and Cornelisen 2003). These previous results were interpreted in relation to the DBL which is assumed to be thinner under oscillatory flow compared to unidirectional flow of the same average speed, resulting from the increased frequency of the disruptions of the boundary layer gradients formed at the blade surface by increasing turbulence (Denny 1988).

As indicated previously, the results from the study suggest that the DBL may play a significant role in influencing nitrate‐uptake rates and growth rates of juvenile *L. digitata* through the effects of mass‐transfer limitation when nutrient demand is high. However, the ambient water velocities associated with such effects were low, being <5–10 cm · s^−1^. The majority of coastal locations in temperate areas where extensive kelp forests are typically found are high energy environments characterized by variable high velocities associated with wave activity. In general, juvenile kelps in these areas could be expected to show minimal effect of current velocity on growth and nutrient‐uptake rates. However, even in these areas low flow rates may be experienced by macroalgae during slack tide conditions, in rock pools or embayments or during periods of low wave activity. In addition, juvenile plants of *L. digitata* are strongly associated with the understorey of the dense canopy kelp bed where low velocities (5–10 cm · s^−1^) can be experienced (Kregting et al. 2011). Equivalent scenarios may also be found for macroalgal species growing in low (<1 μM) seawater nutrient concentrations where the metabolic demand exceeds the nutrient supply so that decreased DBL thickness associated with either increased flow velocity or exposure to oscillatory motion will have a positive effect on growth rate, as seen for juvenile *L. digitata* in this study and the kelp *M. pyrifera* (Hepburn et al. 2007, Stephens and Hepburn 2014).

## Conclusion

These results demonstrate that when sufficient nitrogen is available seawater to saturate metabolic demand, the type of water motion is unlikely to influence the growth rate of juvenile *L. digitata*. However, in summer when the demand for sustained growth exceeds the supply of nutrients to the blade surface under low flow conditions (<5–10 cm · s^−1^), this may affect the growth rate of the macroalgae and, the productivity. It is reasonable to assume that the rate of nutrient‐uptake under the low flow conditions is controlled by the dynamics of the DBL. Oscillatory motion significantly increased the rate of nitrate‐uptake rate by the blades presumably associated with the reduced thickness of the DBL at the blade surface resulting from increased turbulence adjacent to the surface. The disconnection between increased nitrate‐uptake due to oscillatory flow and growth raises further questions about the scales that drive mass‐transfer limitation of productivity in kelp beds and the influence of waves. Data presented here suggest that larger, bed scale processes rather than those at the blade surface may actually drive the connection between water motion, mass transfer and kelp productivity.

The authors thank P. Johnson, R. Ferrier and A. Werner for providing technical support and expertise in the field and laboratory. The work described in this paper was produced as part of SuperGen Marine Energy Research Consortium II, which was funded by the UK Engineering and Physical Science Research Council (grant number EP/E040136/1).
